# Resveratrol regulates Hsp60 in HEK 293T cells during activation of SIRT1 revealed by nascent protein labeling strategy

**DOI:** 10.29219/fnr.v66.8224

**Published:** 2022-04-21

**Authors:** Tian Su, Zhen Wang, Zhengyi Zhang, Zhanwu Hou, Xiao Han, Fei Yang, Huadong Liu

**Affiliations:** Center for Mitochondrial Biology and Medicine, The Key Laboratory of Biomedical Information Engineering of Ministry of Education, School of Life Science and Technology, Xi’an Jiaotong University, Xi’an, Shaanxi, China

**Keywords:** Hsp60, nascent proteome, resveratrol, SIRT1, transcriptome

## Abstract

**Background:**

Resveratrol, a well-known natural compound and nutrient, activates the deacetylation ability of SIRT1, demonstrating p53-dependent apoptosis functions in many diseases. However, the nascent proteomic fluctuation caused by resveratrol is still unclear.

**Objective:**

In this study, we investigated the effect of resveratrol on the nascent proteome and transcriptome initiated by SIRT1 activation, and we explored the mechanism of resveratrol in HEK 293T cells.

**Methods:**

Bioorthogonal noncanonical amino acid tagging (BONCAT) is a method used to metabolically label nascent proteins. In this strategy, L-azidohomoalanine (AHA) was used to replace methionine (Met) under different conditions. Taking advantage of the click reaction between AHA and terminal alkyne- and disulfide-functionalized agarose resin (TAD resin), we were able to efficiently separate stimulation responsive proteins from the pre-existing proteome. Resveratrol responsive proteins were identified by Liquid Chromatograph-Mass Spectrometer/Mass Spectrometer (LC-MS/MS). Furthermore, changes in mRNA levels were analyzed by transcriptome sequencing.

**Results:**

Integrational analysis revealed a resveratrol response in HEK 293T cells and showed that Hsp60 was downregulated at both the nascent protein and mRNA levels. Knockdown of SIRT1 and Hsp60 provides evidence that resveratrol downregulated Hsp60 through SIRT1 and that Hsp60 decreased p53 through the Akt pathway.

**Conclusions:**

This study revealed dynamic changes in the nascent proteome and transcriptome in response to resveratrol in HEK 293T cells and demonstrated that resveratrol downregulates Hsp60 by activating SIRT1, which may be a possible mechanism by which resveratrol prevents p53-dependent apoptosis by regulating Hsp60.

## Popular scientific summary

- Dynamic changes in the nascent proteome were identified by newly synthesized protein labeling.
- Most genes enriched in the cell cycle and cancer pathway were downregulated under resveratrol stimulation.
- Resveratrol downregulates Hsp60 through SIRT1 activation.
- Hsp60 directly influences p53 expression or through the Akt pathway.

Resveratrol (3,4,5-trihydroxystilbene) participates in the regulation of biological processes in cells and is one of the most widely studied cancer prevention compounds and nutrients (1, 2) that affords protection against several types of cancer (3). Resveratrol is a natural and nonflavonoid polyphenolic compound commonly found in plants, such as grapes, mulberries, and pomegranates. Previous studies have shown that resveratrol induces autophagy through the cAMP signaling pathway in hepatic steatosis models (4). In diabetes mellitus type 2 patients and obese individuals, resveratrol reduces oxidative stress (5), possibly by inhibiting the production of reactive oxygen species (ROS). Additionally, resveratrol plays an antioxidative role by stimulating the expression of nuclear erythroid 2-related factor (6) and reducing myocardial apoptosis induced by hydrogen peroxide through autophagic flux (7). In airway disease, resveratrol demonstrates anti-inflammatory activity by activating peroxisome proliferator-activated receptors. It has been reported that resveratrol causes p53-dependent apoptosis (8) and Cdc2-tyr15 phosphorylation for DNA damage in ovarian cancer cells (1). Resveratrol also affects mitochondrial metabolism and homeostasis (9, 10), and it prolongs the lifespan by activating SIRT1 and peroxisome proliferator-activated receptor-γ coactivator-1α (PGC1-α) in metabolic disease (11). Therefore, studying the response of cells to resveratrol helps to understand the mechanism of resveratrol.

Resveratrol plays different roles in distinct biological processes, and the well-recognized cellular function of resveratrol is SIRT1 activation (12). Binding with resveratrol may enhance the activity of SIRT1 by allosteric regulation and increase its affinity for nicotinamide adenine dinucleotide (NAD^+^) and acetylated subunits. SIRT1 belongs to the sirtuin family and is one of the most well-known proteins related to longevity (13, 14). Experiments in mice have demonstrated that calorie restriction increases SIRT1 activity and extends life (13). In addition, SIRT1 is also related to other biological functions. At the cellular level, SIRT1 regulates DNA repair through the acetylation status of Ku70 (15). Activation of SIRT1 by resveratrol inhibits hypoxia-induced apoptosis by regulating forkhead box O1 (FoxO1) (16), decreases acetylation, and increases the activity of PGC-1α to induce genes for oxidative phosphorylation and mitochondrial biogenesis (9). Deletion of SIRT1 in hepatocytes causes a reduction in fatty acid β-oxidation and leads to inflammation (17). SIRT1 also represses PTP1B transcription and affects insulin resistance (18). Previous studies have indicated that SIRT1 protects against Alzheimer’s disease by interfering with the generation of β-amyloid peptides (19). SIRT1 demonstrates anti-aging activity in mammals and suppresses tumors in cancer associated with aging and metabolic syndrome (20). In the prevention of type 2 diabetes, SIRT1 has become a novel therapeutic target owing to its effects on insulin resistance (21). As a conserved NAD^+^ deacetylase, SIRT1 causes changes in various acetylation modifications of histones or transcription factors. However, systematic investigation of the nascent proteome on the cell response to resveratrol during SIRT1 activation is still lacking.

Bioorthogonal noncanonical amino acid tagging (BONCAT) is a new technique used for labeling a variety of molecules based on the principle of bioorthogonal metabolism and has recently been used to tag and identify or visualize newly synthesized proteins (22). L-azidohomoalanine (AHA) is an amino acid analogue containing an azide group, which is an effective substitute for methionine and is accepted as a substrate by methylthioamide tRNA synthetase (22, 23). At the same time, the presence of AHA is nontoxic and does not affect the rate of protein synthesis or degradation (23). When AHA is added to medium to culture cells without methionine, it is present in the newly synthesized protein. In our previous study, we developed a terminal alkyne- and disulfide-functionalized agarose (TAD) resin that efficiently enriches the AHA-labeled proteome by click reaction (24). Following MS analysis, we quantified the nascent proteome and verified these results by western blot and molecular biology experiments. This AHA-based labeling method is helpful to understand the changes in the nascent proteome during SIRT1 activation by resveratrol.

The aim of this study was to investigate the nascent proteomic effect of resveratrol. We used the AHA-based technique to systematically reveal the changes in the nascent proteome. In addition, RNA sequencing was performed to explore changes in the transcriptome under resveratrol stimulation. To integrate the nascent proteome with the transcriptome, we revealed dynamic changes in cells in response to resveratrol, which helped to determine the resveratrol mechanism.

## Methods and materials

### Reagents

Resveratrol (R107315-25 g), DTT (D104860-25 g), iodoacetamide (IAA, I105563-25 g), and 4-pentynoic acid (P133515-5 g) were purchased from Aladdin (https://www.aladdin-e.com/). Tris(2-carboxyethyl) phosphine hydrochloride (TCEP, C4706-2 g) was purchased from Sigma–Aldrich. TFA and protease cocktail (P8340-1ML) were purchased from Sigma–Aldrich. TRIzol (9109) and Prime Script RT Master Mix (Perfect Real Time) (RR036A) were purchased from Takara. A bicinchoninic acid (BCA) kit was purchased from Thermo Scientific (#23225). Horse radish peroxidase (HRP)-streptavidin was purchased from Sigma–Aldrich (#RABHRP3). SIRT1 (IF3) mouse mAb (Cell Signaling Technology [CST], #8469S), acetylated-lysine (Ac-K-100) (CST, #6952S), p53 (CST, #2524S), and Hsp60(D6F1) XP^®^ rabbit mAb (CST, #12165S) antibodies were purchased from CST. GAPDH antibody (Santa Cruz Biotechnology, sc-47724) was purchased from Santa Cruz Biotechnology. Akt antibody (Abmart, T55561) was purchased from Abmart.

### Cell culture and cell viability assay

HEK 293T cells, acquired from ATCC (Manassas, VA), were cultured in H-DMEM supplemented with 10% fetal bovine serum (FBS), 100 U/mL penicillin, and 100 μg/mL streptomycin. Cells were treated with 20 μM resveratrol for 0, 6, 24, 48, and 72 h. To detect the acetylation level, 1 μM trichostatin A (TSA) was used to treat cells for 6 and 24 h as a positive control (25). Proteins were then extracted. All cells were maintained at 37°C in a 5% CO_2_ humidified environment.

HEK 293T cells were seeded in 96-well plates at a density of 2 × 10^4^ per well for 24 h. Cells were treated with resveratrol at concentrations of 0, 0.2, 0.5, 5, 10, 20, 30, 40, and 50 μM for 4 h. Cell viability was then determined using a 3-(4,5-dimethyl-2-thiazolyl)-2,5-diphenyl-2-H-tetrazolium bromide (MTT) assay (26).

### Newly synthesized protein labels, click reactions, and protein enrichment

HEK 293T cells were cultured in H-DMEM medium. When grown to 90% confluency, cells were starved with D0422 medium supplemented with 10% FBS, 0.584 g/L L-glutamine, and 0.0626 g/L L-cysteine for 2 h. Cells were then washed three times with PBS and cultured with D0422 medium supplemented with 10% FBS, 0.584 g/L L-glutamine, 0.0626 g/L L-cysteine, 20 μM resveratrol, and 4 mm AHA for 4 h. Control cells were cultured with DMEM supplemented with 10% FBS, 0.584 g/L L-glutamine, 0.0626 g/L L-cysteine, and 4 mm L-methionine for 4 h. After treatment, cells were washed three times with PBS.

We prepared the following buffers: cell lysis buffer, containing 8 M urea, 50 mM Tris-HCl (pH = 7.4), 2% protease cocktail (v/v), 1% Triton X-100, 1 mM C_3_H_7_Na_2_PO_6_, 1mM Na_4_O_7_P_2_, 1 mM NaF, and 1 mM Na_3_VO_4_; precipitation buffer, containing acetone (v): absolute ethanol (v): glacial acetic acid (v) = 50: 50: 0.1; resuspension buffer, containing 8 M Urea in 100 mM NH_4_HCO_3_; and click buffer, containing TCEP stock solution (4 mM), CuSO_4_ stock solution (4 mM), and the tert-butyl 2,2,2-trichloroacetimidate (TBTA) stock solution (100 μM).

Cells were suspended in cell lysis buffer supplemented with PMSF (cell lysis buffer: PMSF = 100:1), placed on ice for 30 min, sonicated with a probe sonicator for 180 s at 400 W, and centrifuged for 10 min at 12,000 g. Five volumes of precipitation buffer were added to the sample and incubated at −20°C for 2 h or overnight. The solution was then centrifuged for 20 min at 20,000 r at 4°C. The precipitate was washed with ice-cold acetone and 75% ice-cold ethanol followed by centrifugation for 5 min at 20,000 r at 4°C. The sample was placed at room temperature (RT) for 10–15 min and resuspended in 8 M urea in 100 mM NH_4_HCO_3_. A BCA assay was used to determine the protein concentration.

Some samples were incubated in click buffer with 2 mM alkynylated biotin at RT for 18 h under constant agitation (Eppendorf mixer, 700 rpm/m), and loading buffer was then added for western blotting. Other samples were incubated with TAD resin at RT for 18 h under constant agitation (Eppendorf mixer, 700 rpm/m) for MS samples.

Urea (8 M) and NH_4_HCO_3_ (50 mM) were added to solutions to cleave proteins from TAD resin. Digestion was performed using an in-solution digestion protocol. Samples were desalted, and the concentrations were determined by C18 reversed-phase solid-phase extraction (SPE).

### LC–MS/MS detection

Proteomic data were acquired by QE-plus MS using the data-dependent acquisition (DDA) mode and analyzed by MaxQuant. For each sample, a 5 µL sample was injected and analyzed at a 1 h gradient. The scheduled parallel reaction monitoring (PRM) method was performed with a quadrupole isolation window of 2 m/z units, an automatic gain control target of 1 × 10^6^ ions, a maximum fill time of 120 ms, and an orbitrap resolving power of 35,000 at 200 m/z (27). Collision-induced dissociation products of precursors were detected in the positive ion PRM mode (28). Peptides were identified and quantified by MaxQuant software using the human database downloaded from UniProt.

### Transfection

To generate a cell line stably expressing survivin, HEK 239T cells were transfected with a SIRT1 overexpression plasmid, a SIRT1 knockdown plasmid, and a Hsp60 knockdown plasmid using Lipofectamine reagent for 48 h and selected in medium containing 2 μg/mL puromycin. The primer pairs were as follows for SIRT1 overexpression:

5’-CTAGCTAGCACCATGGCGGACGAGGCGGCCCT (forward) and 5’-TTGGCGCGCCTCATGATTTGTTTGATGGATAGT (reverse).

### RNA extraction, RNA sequencing, and gene expression analysis

Total RNA of HEK 293T cells was extracted using TRIzol and subsequently reverse transcribed into cDNA using PrimeScript RT Master Mix. RNA was sent to Annoroad Company for RNA sequencing. RNA from knockdown cells was used to detect the expression of SIRT1 and Hsp60. Real-time quantitative Polymerase Chain Reaction (RT-PCR) was performed using a Bio-Rad system SYBR Green protocol. The results were analyzed using the 2^–∆∆Ct^ method with actin as an internal reference.

### Western blot assay

Protein samples were separated by 10 or 15% SDS polyacrylamide gel electrophoresis (SDS–PAGE) with a constant voltage of 90 V for 120 min and transferred to nitrocellulose filter membranes at 300 mA for 90 min. The membranes were blocked with 5% skimmed milk in Tris buffered saline with Tween (TBST) buffer for 1 h at RT. The membranes were washed with TBST and then incubated with the following primary antibodies in TBST buffer containing 1% Bovine Serum Albumin (BSA) at 4°C overnight: SIRT1 (1:1000, CST, #8469S), acetylated lysine (Ac-K-100) (1:1000, CST, #6952S), HRP-streptavidin (1:1000, Sigma, #RABHRP3), GAPDH (1:2500, Santa Cruz Biotechnology, sc-47724), Hsp60 (1:1000, CST, #12165S), p53 (1:1000, CST, #2524S), and Akt (1:1000, Abmart, T55561). Following incubation with the primary antibody, the membranes were washed three times, and the secondary antibody (anti-rabbit or anti-mouse IgG at 1:3000 in TBST containing 1% BSA) was cultured with the membranes for 2 h at RT. The membranes were washed three times with TBST, and the western blots were developed using an enhanced chemiluminescence (ELC) western blot detection kit. The intensity of the bands was quantified using ImageJ software. The western blots shown in the figures represent the best results from repeated individual experiments.

### Statistical analysis

The SPSS 25.0, Microsoft Office Excel, Skyline (Ver. 21.1), and GraphPad Prism 5 statistical software packages were used for all statistical analyses. For significant differences, significance (*P* < 0.05) was determined using one-way ANOVA followed by Tukey’s *post hoc* multiple comparisons test unless otherwise stated. The STRING database (https://www.string-db.org/), Cytoscape, and R software were used to plot networks and heatmaps. According to the expression level [Fragments per Kilobase per Million Mapped Fragments (FPKM) value] of the differentially expressed genes in each sample, log based on 2 was utilized, and the Euclidean distance was calculated. The hierarchical cluster method was then used to obtain the overall clustering of the samples. All experiments were performed in triplicate.

## Results

### Resveratrol decreases the histone acetylation level

**Fig. 1:**
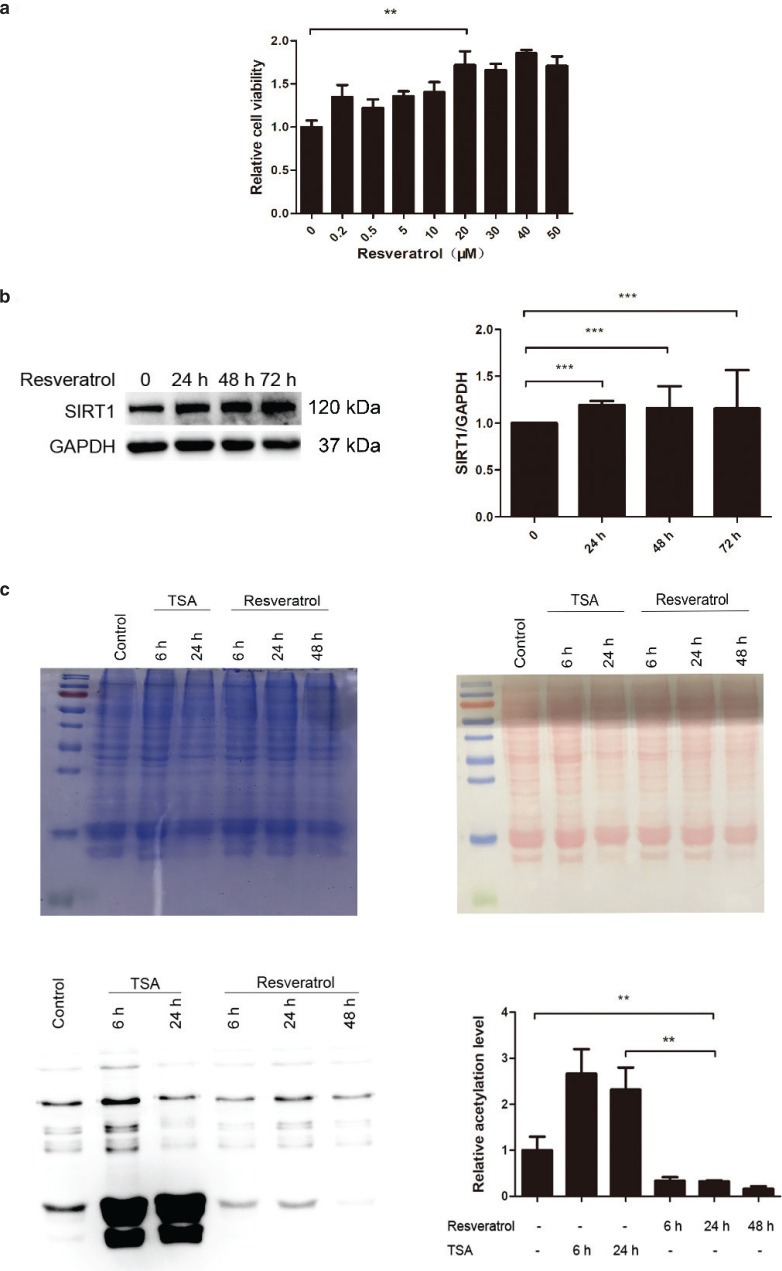
Effects of resveratrol on cell viability, SIRT1 expression, and global acetylation in HEK 293T cells. (a) Cell viability was detected by MTT assay. (b) Resveratrol increased SIRT1 protein levels after resveratrol treatment. (c) Resveratrol decreased global acetylation. The pooled data are shown here as the mean ± SEM, and the significant differences compared to control cells are shown by *P* < 0.01 (**) and *P* < 0.001 (***).

To determine the functionality of resveratrol, we optimized the resveratrol concentration by evaluating cell viability at different time points. HEK 293T cells were treated with resveratrol for 4 h at various concentrations. As shown in Fig. 1a, the MTT assay indicated that resveratrol increased cell viability in a dose-dependent manner. Resveratrol did not cause dramatic damage in cells even at a relatively high concentration (50 μM). Resveratrol at a concentration of 20 μM was used in subsequent experiments unless otherwise noted. Consistent with a previous report, SIRT1 protein levels were increased by resveratrol treatment (Fig. 1b), suggesting that resveratrol successfully regulates SIRT1 in HEK 293T cells. We next investigated whether resveratrol-activated SIRT1 affects global acetylation using TSA as a positive control. Figure 1c clearly shows that the global acetylation level was dramatically reduced, indicating the activation of SIRT1. The change in acetylation was observed as early as 6 h, which is consistent with a previous report (29). Our data indicated that the global acetylation deduction effect lasted for more than 48 h.

### BONCAT strategy reveals the resveratrol-responsive proteome

**Fig. 2:**
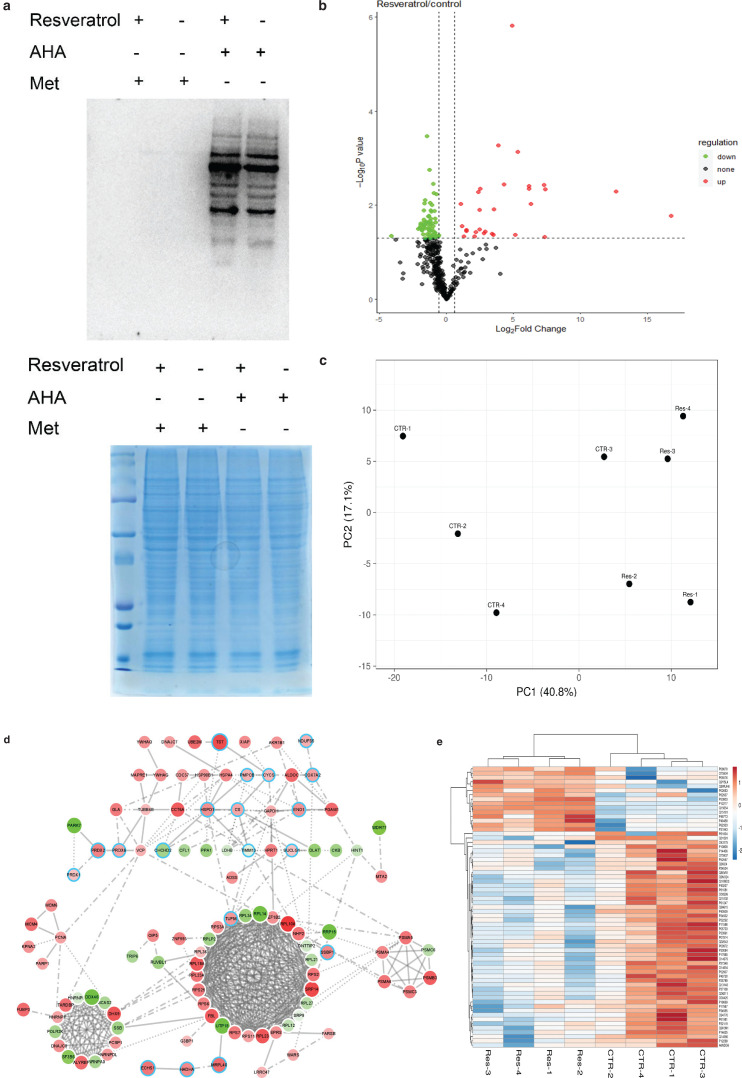
Effects of resveratrol on the nascent proteome. (a) Western blotting for streptavidin horseradish peroxidase (HRP). (b) Volcano plot showing newly synthesized protein with significant changes in mass spectrometry results. The abscissa is the logarithm of the fold change in differential protein expression in the comparison group. Each point represents a specific protein. Significantly upregulated proteins are labeled with red, and significantly downregulated proteins are labeled with green. Proteins that were not significantly differentially expressed are labeled with black. (c) PCA-SVD with imputation was used to calculate principal components. CTR: control. Res: resveratrol. (d) Dramatic changes in nascent proteins were visualized by STRING database. The cutoff was set as 1.5-fold change and *P* < 0.05. The nascent protein level reduced by resveratrol is shown in red, and the nascent protein level increased by resveratrol is shown in green. The size of the circle indicates the fold change, and the large circle indicates a higher fold change. Mitochondrial or redox proteins are highlighted in blue. (e) Heatmap of nascent proteins quantified from cells with different treatments.

Using the BONCAT strategy (30), we obtained proteins labeled by AHA which were separated from the whole proteome subsequently. AHA-labeled proteins directly reflect the changes in protein synthesis when responding to resveratrol treatment. To determine whether AHA is incorporated into the newly synthesized proteins, cell lysates were subjected to a click reaction with alkynylated biotin. Samples were separated by sodium dodecyl sulfate polyacrylamide gel electrophoresis (SDS-PAGE) and subsequently detected by western blot analysis using streptavidin-HRP to visualize the biotin-labeled AHA-containing proteins (Fig. 2a). Cells treated with methionine-containing medium were included as a negative control. The results showed clear bands in AHA-labeled samples, but bands were not observed in control samples. Consistent with previous reports, no dramatic change in nascent protein expression level was observed between resveratrol treatments and dimethyl sulfoxide (DMSO) controls according to the western blot analysis, which may be due to the poor separation of one-dimensional SDS–PAGE for whole cell lysates.

To identify nascent proteins, an enrichment procedure for AHA-labeled proteins is required. Using the TAD resin, we enriched the AHA-labeled proteome of resveratrol-treated cells and performed high-resolution mass spectrometry detection. According to the cutoff at fold-change >1.5, and *P* < 0.05, 29 proteins were upregulated (in red), and 50 proteins were downregulated (in green). The majority did not show significant changes (in gray) (Fig. 2b). PCA revealed that resveratrol-treated nascent proteins were clustered together in comparison with controls (Fig. 2c).

To elucidate the functional relationship among resveratrol-responsive proteins, highly confident interactions were extracted from the STRING database and visualized using Cytoscape (Fig. 2d). Proteins that increased during resveratrol stimulation are represented by green circles, and proteins that decreased during resveratrol stimulation are represented by red circles. Deeper colors indicate large changes. Interestingly, approximately one-third of the proteins plays roles in the mitochondria or redox system, and most of the proteins were located in the top cluster (Fig. 2d). The expression of these resveratrol-responsive mitochondrial proteins was decreased. For example, the expression of stress-responding proteins, such as heat shock protein family D (Hsp60) member 1 (HSPD1), citrate synthase (CS), and alpha-enolase (ENO1), was decreased. In contrast, the TIMM13 mitochondrial intermembrane chaperone, which participates in the import and insertion of multipass transmembrane proteins into the mitochondrial inner membrane, and the CHCHD2 oxygen responsive element-binding protein were upregulated. All these proteins participate in the mitochondrial protein import. The largest cluster mainly composed of ribosome-related proteins, such as the RPL and RPS families, a class of ribosome-related proteins involved in mRNA binding and translational regulation. Components of the proteasome, such as PSMC6, PSMC3, PSMA5, and PSMA6, were clustered into a small group, and these proteins play an important role in either ATP-dependent degradation systems or proteasome complexes. Most of these proteins were decreased, indicating a healthy state of cells. Additionally, mRNA splicing proteins, such as DEAD-box helicase 46 (DDX46) and splicing factor 3b subunit 6 (SF3B6), were upregulated. However, other mRNA regulatory proteins, such as DExH-box helicase 9 (DHX9) and TAR DNA-binding protein (TARDBP), were downregulated.

Figure 2e and Supplementary Table 1 show that most of the dramatically changed proteins were downregulated, which suggested that the transcription of these proteins maybe was inhibited. Resveratrol activated deacetylase, which inhibited transcription, thereby reducing the protein expression level. The upregulated proteins may indicate that these proteins are not protected by acetylation but are mediated by other resveratrol response pathways. Together, these data provided details of the protein rearrangement caused by resveratrol.

### Transcriptome changes caused by resveratrol

**Fig. 3:**
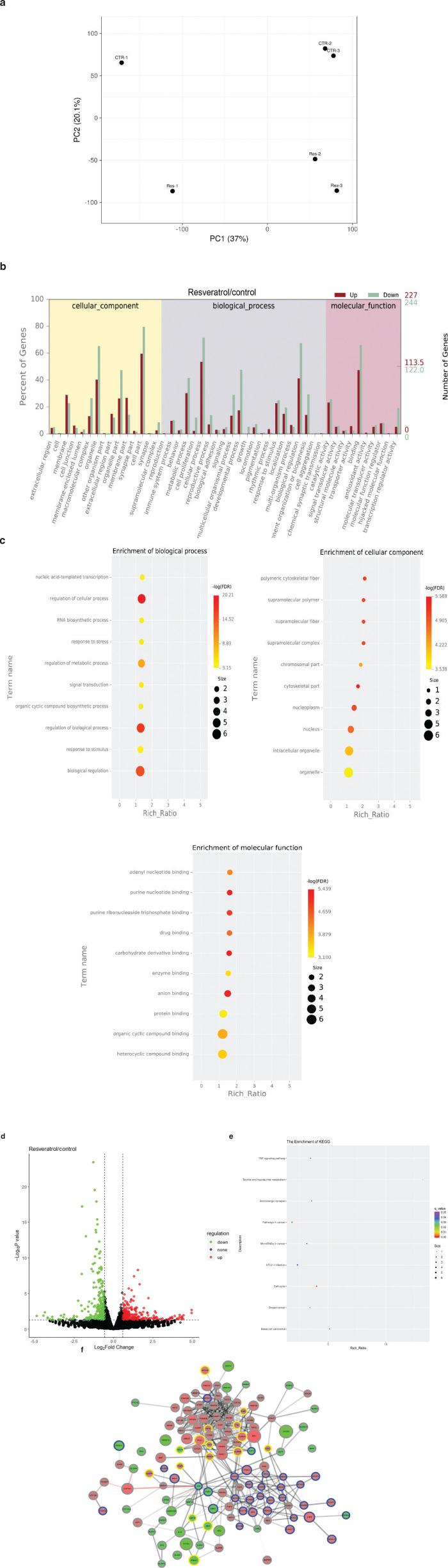
RNA sequencing (RNA-seq) reveals the transcriptome in response to resveratrol. (a) PCA-SVD with imputation was used to calculate principal components. CTR: control. Res: resveratrol. (b) Enrichment of the significantly upregulated or downregulated genes at the secondary GO terms. The abscissa is the GO term. The left ordinate is the percentage of the number of genes, and the right ordinate is the number of genes. (c) GO analysis. The abscissa represents the enrichment ratio, and the ordinate represents different GO items. (d) Volcano plot showing genes with significant changes in RNA sequencing. The abscissa is the logarithm of the fold change in differential protein expression in the comparison group. Each point represents a specific gene. Genes that were significantly upregulated are labeled with red, and genes that were significantly downregulated are labeled with green. Genes that were not significantly differentially expressed are labeled with black. (e) KEGG analysis. The abscissa is the enriching ratio, and the ordinate represents the name of the KEGG pathway. The size of the dot represents the number of differential genes in this pathway, and the color represents the degree of enrichment of the KEGG entry. (f) Genes showing dramatic changes were visualized by STRING database. The cutoff was set as 1.5-fold change and *P* < 0.05. The mRNA level reduced by resveratrol is shown in red, and the mRNA level increased by resveratrol is shown in green. The size of the circle indicates the fold change, and the large circle indicates a higher fold change. Transcriptional regulatory genes are highlighted by blue circles, and the kinases are highlighted by yellow circles.

Cells respond to resveratrol mainly in the following three ways: transcriptome, proteome, and protein posttranslational modifications. Once resveratrol activates SIRT1, the mRNA level changes because acetylation is a hallmark of gene transcription activation. Histone acetylation is one of the key components of the histone code. To quantify the mRNA changes, transcriptome sequencing was performed in triplicate for both the resveratrol and DMSO control treatments. Figure 3a shows that different samples were clustered into two groups according to the treatment, suggesting that the technical repeats were highly reliable. The resveratrol-mediated gene enrichments in biological processes, cellular components, and molecular functions are shown in Fig. 3b and c. Our data clearly showed that most resveratrol-responsive genes were related to organelle parts and cell parts in cellular components, regulation of cellular processes, and biological processes. As shown in Fig. 3b, approximately 70% of downregulated genes and 50% of upregulated genes were involved in cellular processes. The secondary significant process was biological regulation, which included approximately 67.35% of downregulated genes and 41.23% of upregulated genes. Approximately 161 changed genes were functional in binding and catalytic activity (Fig. 3b). Figure 3c shows that most genes were enriched in organelles but had higher *q* values than the others. A few significant genes belonged to the cytoskeletal part. We observed 473 genes that showed significant (*P* < 0.05) changes, including 228 upregulated genes and 245 downregulated genes (Fig. 3d and Supplementary Table 2), which indicated that resveratrol did not cause global inhibition of genes. By using the cutoff at fold change >2 and *P* < 0.05, significantly changed genes were subjected to GO analysis. Additionally, we mapped these genes to the Kyoto Encyclopedia of Genes and Genomes (KEGG) pathways. As shown in Fig. 3e, the top three enriched pathways were cell cycle, cancer, and breast cancer. Among these pathways, most genes were downregulated.

To further understand the relationship of these genes, highly confident protein–protein interactions were extracted from the STRING database and visualized by Cytoscape. As shown in Fig. 3f, the green circles represent upregulated genes, and the red circles represent downregulated genes. In general, there were more decreased proteins than increased proteins. Significantly changed genes were clustered into three groups. Figure 3f shows that the top cluster was mainly composed of increased genes, and they were cell division-related genes, such as proline and serine rich coiled-coil 1 (PSRC1), which are required for normal progression through mitosis. Additionally, PIF1 is required for the maintenance of both mitochondrial and nuclear genome stability. Interestingly, many kinases, such as aurora kinase A (AURKA) and polo-like kinase 1 (PLK1) (marked by yellow circles), were also grouped in this cluster, indicating activated cell cycling. Another large group of increased genes consisted of transcription regulatory-related genes, and they are labeled by blue circles. Transcription activators, such as forkhead box C2 (FOXC2), LIM homeobox 8 (LHX8), and SP8, were significantly upregulated. Stress response-related genes, such as interferon-induced transmembrane protein 3 (IFITM3), growth arrest and DNA damage inducible beta (GADD45B), and activity regulated cytoskeleton associated protein (ARC), were enriched in the decreased cluster. All these data indicated that resveratrol maintained the cells in healthy and stressless states.

### Resveratrol reduces Hsp60 expression

**Fig. 4:**
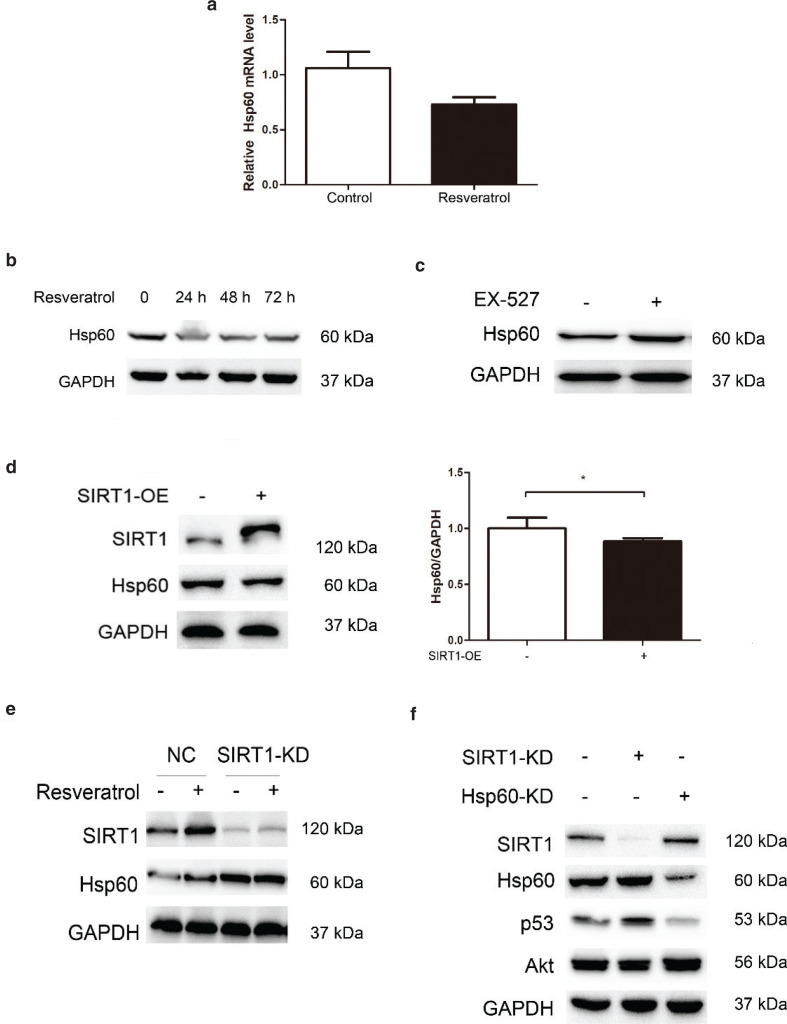
Resveratrol decreases Hsp60 through SIRT1 regulation. (a) The mRNA level of Hsp60 was decreased. (b) Resveratrol decreased Hsp60 expression. (c) Inhibition of SIRT1 activity increased Hsp60 expression. (d) Hsp60 was decreased in SIRT1-OE cells. The pooled data are shown here as the mean ± SEM, and the significant differences compared to the control cells are shown by *P* < 0.05 (*). (e) Hsp60 was increased in SIRT1-KD cells. (f) Effects of knocking down Hsp60 on Akt and p53 protein expression.

Integrated analysis of the nascent proteome and transcriptome showed similar trends after resveratrol treatment. Interestingly, we found that 93 proteins, including heat shock protein 60 (Hsp60), fatty acid synthase (FASN), and catalase (CAT), were increased or decreased at both the transcriptional and translational levels, and Hsp60 was more significant among these proteins. Hsp60 was one of the proteins with consistent changes trending both in the nascent proteome and transcriptome. To confirm the high-throughput data, RT-PCR and western blot analysis were performed. Both the Hsp60 mRNA level (Fig. 4a) and protein level (Fig. 4b) were reduced by resveratrol, and this downregulation caused by resveratrol lasted at least 48 h, suggesting that resveratrol decreases Hsp60 both in the transcriptome and proteome. Because resveratrol is an activator of SIRT1, the acetylation level decreased (Fig. 1c), indicating that SIRT1 functions as a deacetylase. Therefore, we used EX-527 to inhibit SIRT1 activity to determine whether resveratrol decreases Hsp60 through SIRT1 regulation. As shown in Fig. 4c, the Hsp60 protein level was increased after EX-527 treatment, indicating that SIRT1 is involved in Hsp60 expression initiation. Furthermore, we overexpressed SIRT1 in HEK 293T cells and found a reduction in Hsp60 in SIRT1-overexpressing (SIRT1-OE) cells (Fig. 4d). In contrast, Hsp60 was increased in SIRT1-knockdown (SIRT1-KD) cells (Fig. 4e). Together, these data suggested that resveratrol downregulated Hsp60 expression by increasing SIRT1 activity and expression.

**Fig. 5:**
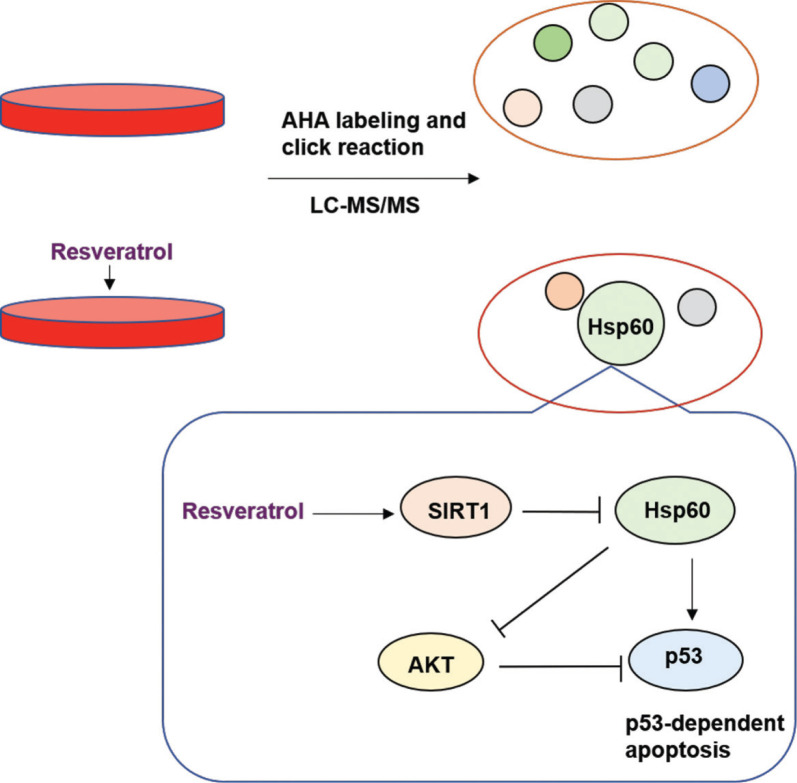
Resveratrol decreases Hsp60 expression via regulation of SIRT1 as revealed by a nascent protein labeling strategy. Resveratrol-induced activation of SIRT1 leads to a reduction in Hsp60. The reduction in Hsp60 influenced Akt and p53 expression, suggesting that Hsp60 affects p53-dependent apoptosis.

To determine what functions resveratrol affects by regulating Hsp60, we knocked down Hsp60 in HEK 293T cells. In Hsp60-knockdown (Hsp60-KD) cells, p53 was reduced (Fig. 4f), and Akt, which is upstream of the p53 pathway, was increased. These results suggested that Hsp60 affects p53 pathway by increasing Akt expression (Fig. 5).

## Discussion

This study defined the effects of SIRT1 activation on the nascent proteome triggered by resveratrol in HEK 293T cells. By using the BONCAT strategy, we labeled the nascent proteome of cells treated with resveratrol and quantified it by LC–MS/MS. In combination with RNA sequencing, we identified 199 proteins responding to resveratrol in both transcription and expression. RNA sequencing presented the whole mRNA level and did not directly correlate with protein translation. Therefore, our research on nascent proteins may better reflect the response of cells to stimulation.

LC–MS/MS detection suggested that most nascent proteins showed a decreasing trend in expression. SIRT1 is a deacetylase that reduces acetylation levels. Therefore, we predicted that the reduction in nascent proteins is at least partially due to the activation of SIRT1 by resveratrol. In this study, we found that MCM6, which is a component of the MCM2-7 complex (MCM complex), decreased in the nascent proteome. SIRT1 regulates DNA binding and the stability of the MCM10 DNA replication factor via deacetylation (31). MCM10 initiates DNA replication and facilitates MCM2-7 helicase binding with DNA polymerase alpha. Therefore, SIRT1 may affect MCM6 via deacetylation. Another protein, the AP-1 transcription factor subunit c-Jun, is a transcription factor that was decreased at the nascent protein level. SIRT1 has been found to inhibit the transcriptional activity of c-Jun (32). These results suggest that resveratrol regulates c-Jun via the activation of SIRT1. In addition, we found other proteins that are transcription factors, and these proteins either have previously been identified to be influenced by SIRT1 or have the potential to be influenced by SIRT1, such as FOXO3, SUV39H1, FOXO1, RUNX2, and SOX2, providing additional information for the link between SIRT1 function and the nascent proteome.

Among these changing proteins, Hsp60 showed a great reduction in nascent protein levels. Hsp60 belongs to heat shock proteins and is a mitochondrial protein. Heat shock proteins refer to a group of proteins produced under cell stress conditions, especially high temperature, and many heat shock proteins are molecular chaperones. For example, Hsp60 and Hsp10 are molecular chaperones that facilitate the correct folding and import of proteins. These proteins also prevent the misfolding of peptides and protein aggregation under stress conditions (33). In addition to MS data, we also observed a reduction in Hsp60 by western blot analysis. However, this change in the whole proteome was not apparent, but significant change was observed in the nascent proteome. There may be other unknown factors that cause the final expression of Hsp60 to be different from that of nascent protein. Our results showed that the activity and expression of SIRT1 influenced Hsp60 expression, but this effect also was obvious due to insignificant changes in the whole proteome of Hsp60.

It has been reported that Hsp60 regulates tumor cell apoptosis (34) and is an apoptosis-related target (33). Hsp60 knockdown induces p53-dependent apoptosis in MCF-7 cells (34). Moreover, Hsp60 physically associates with p53 but Hsp60 has no effect on MDM2 (34), which contradicted our research. Our data provided new evidence to suggest that Hsp60 affects the p53 pathway through Akt signaling in HEK 293T cells. By knocking down Hsp60, we showed that Hsp60 modulates the expression of Akt and p53. Because Akt is upstream of MDM2 and p53, changes in Akt expression may influence the p53 pathway. It is possible that a reduction in Hsp60 affects the p53 pathway through Akt. We cannot completely exclude the possibility that in this experimental system, Hsp60 regulates p53 through another pathway. However, the effects of Hsp60 on Akt and p53 expression were identical.

In conclusion, our findings showed that resveratrol decreases Hsp60 expression in the nascent proteome and transcriptome through SIRT1 activation in HEK 293T cells, which may affect p53 expression through the Akt pathway. This evidence suggests a new mechanism by which resveratrol may influence p53-dependent apoptosis by regulating Hsp60. Therefore, Hsp60 may be a potential target of resveratrol and a tumor marker. Additionally, our strategy provided a model to investigate the depth mechanism of other drug or nutrition. Further investigation into the relationship between resveratrol and the nascent proteome may provide new opportunities to identify the target of resveratrol and provide new strategies for the prevention and treatment of resveratrol in tumors.

## Supporting information

Supporting information is available from the author.

## Supplementary Material

Resveratrol regulates Hsp60 in HEK 293T cells during activation of SIRT1 revealed by nascent protein labeling strategy
