# Supplementary material for: Resveratrol regulates Hsp60 in HEK 293T cells during activation of SIRT1 revealed by nascent protein labeling strategy
Source: Food Nutr Res. 2022 Apr 21;66:10.29219/fnr.v66.8224. doi: 10.29219/fnr.v66.8224 (PMC9034730; doi:10.29219/fnr.v66.8224)
Supplement: Resveratrol regulates Hsp60 in HEK 293T cells during activation of SIRT1 revealed by nascent protein labeling strategy [file FNR-66-8224-s001.docx]

**Supplementary materials**

**Table S1** Differentially expressed nascent proteins identified by LC–MS/MS

| **Protein ID** | **Fold Change (Res/CTR)** | **log2 FC** | **P** | **Regulation** |
| --- | --- | --- | --- | --- |
| P02663 | 2.475471084 | 1.3077031 | 0.046135 | up |
| O75934 | 11.57111648 | 3.5324562 | 0.012396 | up |
| P06576 | 2.834516419 | 1.5031026 | 0.033896 | up |
| P08670 | 2.247727082 | 1.1684669 | 0.028128 | up |
| P10515 | 167.5612437 | 7.3885447 | 0.004618 | up |
| P12277 | 40.19346729 | 5.3288891 | 0.000733 | up |
| P19623 | 5.711007019 | 2.5137452 | 0.004484 | up |
| P23528 | 78.92674763 | 6.3024424 | 0.009456 | up |
| P30050 | 163.7226777 | 7.3551104 | 0.047183 | up |
| P31943 | 5.457183615 | 2.4481566 | 0.01271 | up |
| P49207 | 14.80147376 | 3.8876689 | 0.000541 | up |
| P49458 | 2.785303227 | 1.4778344 | 0.035581 | up |
| P49773 | 11.27007325 | 3.494425 | 0.042415 | up |
| P50914 | 6397.318829 | 12.643252 | 0.005163 | up |
| P53803 | 34.7187646 | 5.1176437 | 0.04272 | up |
| P62333 | 19.80698239 | 4.3079372 | 0.003642 | up |
| P62937 | 4.580032638 | 2.1953579 | 0.037372 | up |
| Q15181 | 71.43702929 | 6.1586002 | 0.004538 | up |
| Q15654 | 29.78220065 | 4.8963785 | 1.53E-06 | up |
| Q5QJE6 | 7.450705584 | 2.8973771 | 0.036949 | up |
| Q8WUH6 | 5.217932607 | 2.3834783 | 0.005296 | up |
| Q99497 | 4.265883079 | 2.0928444 | 0.045799 | up |
| Q9BQA1 | 6.919211181 | 2.7906076 | 0.039057 | up |
| Q9Y265 | 72.10790732 | 6.1720856 | 0.003904 | up |
| Q9Y3B4 | 5.505540552 | 2.4608842 | 0.032629 | up |
| Q9Y3B9 | 110839.754 | 16.758116 | 0.017089 | up |
| Q9Y5J1 | 10.63837078 | 3.4112053 | 0.04018 | up |
| Q9Y5L4 | 2.10604495 | 1.0745362 | 0.009531 | up |
| Q9Y6H1 | 153.9983459 | 7.266771 | 0.003751 | up |
| A6NDG6 | 0.359151915 | -1.477334 | 0.042263 | down |
| P34955 | 0.355701984 | -1.491259 | 0.022918 | down |
| O00299 | 0.322936824 | -1.630676 | 0.009201 | down |
| O00425 | 0.249686053 | -2.001813 | 0.030448 | down |
| O14654 | 0.470200076 | -1.088653 | 0.031779 | down |
| O14979 | 0.471626796 | -1.084282 | 0.047197 | down |
| O43175 | 0.407586488 | -1.294822 | 0.023481 | down |
| O43396 | 0.511182871 | -0.968089 | 0.003529 | down |
| O75390 | 0.501612787 | -0.995354 | 0.028143 | down |
| O75937 | 0.429927769 | -1.217834 | 0.02157 | down |
| O94776 | 0.417084465 | -1.261589 | 0.03702 | down |
| P04406 | 0.544007379 | -0.878302 | 0.013448 | down |
| P06733 | 0.400967551 | -1.318443 | 0.016725 | down |
| P07814 | 0.432535431 | -1.20911 | 0.049866 | down |
| P09874 | 0.528712172 | -0.919446 | 0.048081 | down |
| P09972 | 0.313071585 | -1.675436 | 0.012994 | down |
| P10809 | 0.436037913 | -1.197475 | 0.019271 | down |
| P11586 | 0.379300568 | -1.398587 | 0.023732 | down |
| P12268 | 0.295938474 | -1.756631 | 0.041763 | down |
| P14406 | 0.487542743 | -1.036399 | 0.024212 | down |
| P14625 | 0.487381756 | -1.036876 | 0.045099 | down |
| P17980 | 0.366218142 | -1.449225 | 0.023475 | down |
| P17987 | 0.251246251 | -1.992826 | 0.030492 | down |
| P18669 | 0.309390649 | -1.692498 | 0.027432 | down |
| P24534 | 0.406344316 | -1.299225 | 0.033884 | down |
| P25789 | 0.408676199 | -1.29097 | 0.02813 | down |
| P27348 | 0.433665575 | -1.205345 | 0.009059 | down |
| P30084 | 0.328471307 | -1.606161 | 0.03122 | down |
| P32119 | 0.415987977 | -1.265386 | 0.042457 | down |
| P33991 | 0.33295719 | -1.586591 | 0.0267 | down |
| P34932 | 0.390133652 | -1.35796 | 0.017259 | down |
| P37108 | 0.234527798 | -2.092169 | 0.031741 | down |
| P40227 | 0.295322942 | -1.759635 | 0.030132 | down |
| P40939 | 0.363234902 | -1.461025 | 0.000345 | down |
| P46109 | 0.556483935 | -0.845588 | 0.045231 | down |
| P49720 | 0.279006845 | -1.841628 | 0.033661 | down |
| P52292 | 0.444789602 | -1.168805 | 0.01308 | down |
| P52565 | 0.593251955 | -0.753283 | 0.045426 | down |
| P52597 | 0.478996058 | -1.061914 | 0.042497 | down |
| P52907 | 0.45558178 | -1.134218 | 0.013824 | down |
| P53597 | 0.599896744 | -0.737214 | 0.00591 | down |
| P55072 | 0.513121763 | -0.962627 | 0.005549 | down |
| P61081 | 0.306026199 | -1.708273 | 0.023062 | down |
| P61247 | 0.439548574 | -1.185905 | 0.035027 | down |
| P61604 | 0.056550035 | -4.144328 | 0.045057 | down |
| P61981 | 0.396098937 | -1.336067 | 0.016913 | down |
| P63220 | 0.538124961 | -0.893987 | 0.038977 | down |
| P68371 | 0.556620943 | -0.845233 | 0.019565 | down |
| Q01581 | 0.431174569 | -1.213656 | 0.025127 | down |
| Q02543 | 0.262287492 | -1.930779 | 0.026448 | down |
| Q06830 | 0.670458707 | -0.57678 | 0.043554 | down |
| Q08211 | 0.259990278 | -1.94347 | 0.02476 | down |
| Q13148 | 0.32982223 | -1.600239 | 0.049936 | down |
| Q13283 | 0.588753334 | -0.764265 | 0.046066 | down |
| Q14566 | 0.40871401 | -1.290836 | 0.001761 | down |
| Q15102 | 0.309724403 | -1.690943 | 0.020381 | down |
| Q1KMD3 | 0.414413124 | -1.270858 | 0.047717 | down |
| Q7RTV0 | 0.522473143 | -0.936571 | 0.036629 | down |
| Q86V81 | 0.281535655 | -1.82861 | 0.035057 | down |
| Q8N1G4 | 0.569956133 | -0.811077 | 0.02305 | down |
| Q96I24 | 0.317024356 | -1.657334 | 0.025716 | down |
| Q99615 | 0.475716399 | -1.071826 | 0.010069 | down |
| Q9H3N1 | 0.39075122 | -1.355678 | 0.031072 | down |
| Q9NSD9 | 0.59172186 | -0.757009 | 0.048059 | down |
| Q9NX24 | 0.328184054 | -1.607423 | 0.007787 | down |
| Q9NX63 | 0.64559013 | -0.63131 | 0.046619 | down |
| Q9NZI8 | 0.548977131 | -0.865182 | 0.038944 | down |

**Table S2** Differentially expressed genes identified by RNA-sequencing

| **Gene Name** | **Fold Change (Res/CTR)** | **Log2 FC** | **P** | **Regulation** |
| --- | --- | --- | --- | --- |
| SLC6A13 | 1.747333 | 0.805155 | 0.003127 | up |
| ALOX5 | 1.80915 | 0.855312 | 0.033782 | up |
| NPC1L1 | 1.673688 | 0.743031 | 0.010265 | up |
| SLC7A9 | 2.785812 | 1.478098 | 0.045476 | up |
| CYP46A1 | 5.252926 | 2.393121 | 0.027786 | up |
| SOX30 | 1.861622 | 0.89656 | 0.029987 | up |
| TG | 2.328834 | 1.219608 | 0.015826 | up |
| ZNF280C | 1.534246 | 0.61753 | 0.002877 | up |
| CYBRD1 | 1.573141 | 0.653648 | 0.017985 | up |
| CASS4 | 1.647632 | 0.720394 | 0.000454 | up |
| NLRC4 | 1.634076 | 0.708475 | 0.004601 | up |
| IZUMO4 | 1.869614 | 0.90274 | 0.03523 | up |
| GADD45B | 2.153592 | 1.106745 | 7.17E-05 | up |
| GGT1 | 1.67334 | 0.74273 | 0.000116 | up |
| SGK2 | 2.555288 | 1.353486 | 0.009337 | up |
| CRISPLD2 | 2.91036 | 1.541198 | 4.88E-09 | up |
| AP3B2 | 1.506713 | 0.591405 | 0.011679 | up |
| JAK3 | 2.003339 | 1.002407 | 0.016186 | up |
| LFNG | 1.858554 | 0.894181 | 0.043443 | up |
| NSUN5P2 | 1.688466 | 0.755713 | 0.024972 | up |
| RASSF4 | 1.647971 | 0.720691 | 0.026101 | up |
| SEPTIN4 | 2.456427 | 1.296562 | 2.65E-05 | up |
| RASD1 | 2.223546 | 1.152862 | 0.002005 | up |
| SLC6A4 | 1.853398 | 0.890173 | 0.044705 | up |
| TECTA | 1.850615 | 0.888005 | 0.002958 | up |
| SLC6A12 | 2.608551 | 1.383249 | 0.002354 | up |
| C12orf57 | 2.069844 | 1.049522 | 0.001055 | up |
| ST8SIA1 | 4.009943 | 2.003582 | 0.023537 | up |
| E2F3 | 1.557922 | 0.639623 | 0.000548 | up |
| PTCH2 | 1.604677 | 0.682283 | 0.002364 | up |
| ZC2HC1C | 1.779805 | 0.831719 | 0.038543 | up |
| SEPT7P9 | 5.907176 | 2.562469 | 0.025895 | up |
| EGR1 | 1.795202 | 0.844146 | 0.000156 | up |
| EPX | 1.831176 | 0.87277 | 0.006438 | up |
| TEX14 | 1.554252 | 0.636221 | 0.001111 | up |
| RAB9B | 1.891598 | 0.919606 | 0.041273 | up |
| SNAI1 | 1.942318 | 0.95778 | 0.001271 | up |
| MMP24 | 2.587869 | 1.371765 | 0.021896 | up |
| ZFP36 | 1.609019 | 0.686181 | 0.010576 | up |
| IGLL1 | 13.09568 | 3.711019 | 0.030012 | up |
| VGF | 1.949336 | 0.962983 | 0.034758 | up |
| IRF5 | 2.518861 | 1.332772 | 0.038937 | up |
| TPH1 | 1.791352 | 0.841049 | 0.003925 | up |
| KIAA1683 | 2.577873 | 1.366181 | 0.008331 | up |
| POLN | 1.626783 | 0.702021 | 0.006883 | up |
| ZNF132 | 1.654061 | 0.726013 | 0.001731 | up |
| SYT11 | 1.589887 | 0.668924 | 0.044079 | up |
| C1QTNF6 | 1.624847 | 0.700304 | 1.36E-05 | up |
| ACRV1 | 2.585426 | 1.370402 | 0.021906 | up |
| BAAT | 1.603715 | 0.681418 | 0.003914 | up |
| N4BP2L1 | 1.713757 | 0.777162 | 0.016426 | up |
| DNAJA4 | 1.974491 | 0.98148 | 0.008865 | up |
| CARD14 | 1.714645 | 0.77791 | 0.031441 | up |
| ACP4 | 1.50597 | 0.590693 | 0.036335 | up |
| C1orf162 | 2.724798 | 1.446149 | 0.008164 | up |
| PLEKHA6 | 1.951789 | 0.964797 | 0.00547 | up |
| RHOB | 1.612874 | 0.689634 | 0.001789 | up |
| LGSN | 2.524352 | 1.335913 | 0.001332 | up |
| PNLDC1 | 3.268857 | 1.708786 | 0.005315 | up |
| WDR31 | 1.523634 | 0.607516 | 0.022308 | up |
| GAS2 | 2.152241 | 1.105839 | 0.039578 | up |
| CCDC102B | 2.164658 | 1.114139 | 0.037417 | up |
| MMP14 | 1.527045 | 0.610743 | 0.046671 | up |
| KCNJ6 | 2.18331 | 1.126517 | 0.03896 | up |
| LCA5L | 1.520692 | 0.604728 | 0.040481 | up |
| PKD1L1 | 1.985556 | 0.989543 | 0.011334 | up |
| ADGRG5 | 2.750854 | 1.459879 | 0.029619 | up |
| NPR2 | 2.351218 | 1.233409 | 0.025386 | up |
| CPAMD8 | 2.85154 | 1.511741 | 0.025314 | up |
| PAQR6 | 1.591205 | 0.67012 | 0.009033 | up |
| PPP1R32 | 1.66353 | 0.734248 | 0.00863 | up |
| WDR64 | 1.672603 | 0.742095 | 0.03074 | up |
| IL17RE | 1.842623 | 0.881761 | 0.025731 | up |
| SLC45A2 | 1.900416 | 0.926315 | 0.006786 | up |
| MYOZ3 | 3.482236 | 1.800014 | 0.009532 | up |
| PRSS37 | 2.731496 | 1.449691 | 0.023948 | up |
| AMER2 | 2.928982 | 1.550399 | 0.021944 | up |
| IFI27 | 1.562615 | 0.643962 | 0.016904 | up |
| RRAD | 2.360212 | 1.238917 | 0.031719 | up |
| TTYH1 | 1.563325 | 0.644618 | 0.018163 | up |
| PLIN4 | 1.752361 | 0.8093 | 0.002019 | up |
| VASN | 2.189614 | 1.130676 | 3.46E-06 | up |
| MFSD7 | 1.673553 | 0.742914 | 0.016249 | up |
| C8orf46 | 1.827723 | 0.870047 | 0.000399 | up |
| IL13 | 2.192881 | 1.132827 | 0.001845 | up |
| PTAFR | 2.369417 | 1.244532 | 0.017908 | up |
| ANKK1 | 6.620759 | 2.726997 | 0.020333 | up |
| FOS | 2.367294 | 1.243239 | 0.000112 | up |
| LRRN2 | 1.638271 | 0.712174 | 0.015966 | up |
| SERPINB9 | 1.593076 | 0.671815 | 0.004373 | up |
| ZNF160 | 1.639311 | 0.71309 | 0.008812 | up |
| FBXW10 | 1.635375 | 0.709622 | 0.018884 | up |
| SLC22A1 | 2.366874 | 1.242983 | 0.00978 | up |
| BAIAP2 | 1.522574 | 0.606512 | 0.000255 | up |
| TSPEAR | 1.541865 | 0.624676 | 0.01642 | up |
| PLEKHD1 | 6.849812 | 2.776064 | 0.037735 | up |
| ZNF843 | 1.978674 | 0.984534 | 0.005326 | up |
| MIR7-3HG | 4.613794 | 2.205954 | 4.57E-05 | up |
| EFCAB5 | 4.557227 | 2.188156 | 0.01169 | up |
| EPS8L2 | 1.540896 | 0.623769 | 0.024519 | up |
| JUN | 1.546508 | 0.629015 | 4.99E-05 | up |
| ZNF454 | 20.0985 | 4.329016 | 0.018353 | up |
| GPR35 | 2.536272 | 1.34271 | 0.038047 | up |
| LINC00324 | 14.69964 | 3.877709 | 0.014431 | up |
| ZFP3 | 12.1552 | 3.603502 | 0.031762 | up |
| C17orf47 | 18.24311 | 4.18928 | 0.010649 | up |
| UBA7 | 1.685699 | 0.753347 | 0.014487 | up |
| ARHGEF37 | 2.195403 | 1.134486 | 0.001594 | up |
| PTGDR2 | 2.4247 | 1.277806 | 0.044955 | up |
| FAM46C | 1.939182 | 0.955448 | 2.09E-06 | up |
| PGBD2 | 1.779496 | 0.831469 | 0.000967 | up |
| IL3RA | 1.667486 | 0.737675 | 0.015805 | up |
| SOCS1 | 3.12057 | 1.64181 | 0.005024 | up |
| PDE6G | 2.136059 | 1.094952 | 0.008708 | up |
| CCDC73 | 20.11436 | 4.330154 | 0.04429 | up |
| PLCD1 | 1.666234 | 0.736591 | 0.018822 | up |
| HSD3BP1 | 16.35577 | 4.031728 | 0.034797 | up |
| P2RX2 | 1.746957 | 0.804844 | 0.007614 | up |
| SAXO2 | 1.685523 | 0.753196 | 0.002841 | up |
| SBSN | 2.208247 | 1.142901 | 0.022136 | up |
| ANKDD1B | 2.787435 | 1.478938 | 0.001053 | up |
| BECN2 | 4.780768 | 2.257243 | 0.035385 | up |
| C20orf204 | 1.51721 | 0.601421 | 0.025888 | up |
| CHRNG | 3.503744 | 1.808897 | 0.042314 | up |
| TEX43 | 2.692945 | 1.429185 | 0.003471 | up |
| SERTAD1 | 1.644069 | 0.717271 | 0.016411 | up |
| CFAP43 | 1.932596 | 0.95054 | 0.004353 | up |
| NPIPB6 | 1.750054 | 0.807399 | 0.020535 | up |
| ARC | 2.432654 | 1.282531 | 3.92E-08 | up |
| SHISA4 | 1.716657 | 0.779602 | 0.005814 | up |
| RNU5A-1 | 2.243345 | 1.165652 | 0.031031 | up |
| RNU4-23P | 12.13231 | 3.600782 | 0.0284 | up |
| RNU5B-1 | 3.761355 | 1.911252 | 0.034921 | up |
| RNVU1-6 | 2.414616 | 1.271794 | 0.02911 | up |
| DISP3 | 2.413669 | 1.271228 | 0.026163 | up |
| FRG2 | 5.819115 | 2.5408 | 0.013416 | up |
| IGLV1-50 | 3.063244 | 1.61506 | 0.020665 | up |
| LTC4S | 2.944379 | 1.557963 | 0.046386 | up |
| NOTO | 17.99301 | 4.169364 | 0.023372 | up |
| POLR2J4 | 1.758062 | 0.813986 | 0.007385 | up |
| LINC01588 | 1.838461 | 0.878498 | 0.015038 | up |
| IFIT1P1 | 10.17668 | 3.347195 | 0.048493 | up |
| AL354719.1 | 21.18643 | 4.405069 | 0.037855 | up |
| RFPL4A | 2.507104 | 1.326022 | 0.001827 | up |
| NDUFB4P8 | 2.655587 | 1.409031 | 0.027974 | up |
| AL391095.1 | 1.768113 | 0.82221 | 0.029165 | up |
| RAB6C-AS1 | 1.628103 | 0.703192 | 0.046179 | up |
| AL731684.1 | 17.71138 | 4.146605 | 0.0367 | up |
| RUSC1-AS1 | 1.538376 | 0.621408 | 0.002915 | up |
| FRG2B | 2.539035 | 1.34428 | 0.020332 | up |
| AC102953.1 | 30.72221 | 4.94121 | 0.005831 | up |
| IFNA20P | 2.268799 | 1.181929 | 0.008994 | up |
| ITGB2-AS1 | 16.04152 | 4.003739 | 0.042469 | up |
| RBM26-AS1 | 1.835528 | 0.876195 | 0.000258 | up |
| AC138393.2 | 8.503875 | 3.08812 | 0.033559 | up |
| AP000695.1 | 11.07944 | 3.469813 | 0.040252 | up |
| ERVH48-1 | 4.215506 | 2.075706 | 0.049526 | up |
| LINC01687 | 1.953056 | 0.965733 | 0.016593 | up |
| SNRK-AS1 | 5.683629 | 2.506812 | 0.04026 | up |
| AL354719.2 | 6.144223 | 2.619231 | 0.003268 | up |
| LINC00854 | 4.429678 | 2.147202 | 0.005516 | up |
| LINC00106 | 1.801488 | 0.849189 | 0.040203 | up |
| ANO7L1 | 2.059523 | 1.04231 | 0.011217 | up |
| ACA64 | 12.99426 | 3.699803 | 0.02097 | up |
| AMY2B | 1.730555 | 0.791235 | 0.018818 | up |
| AL132838.1 | 6.020029 | 2.58977 | 0.035548 | up |
| AL512306.2 | 2.096625 | 1.068069 | 0.000144 | up |
| UOX | 1.778722 | 0.830841 | 0.008905 | up |
| AL512306.3 | 2.311827 | 1.209033 | 0.048879 | up |
| WDR82P2 | 6.341928 | 2.664922 | 0.024901 | up |
| RTL5 | 1.60296 | 0.680739 | 0.007979 | up |
| AC006387.1 | 10.99598 | 3.458904 | 0.048914 | up |
| GABPB1-AS1 | 1.617622 | 0.693875 | 0.000398 | up |
| AC022126.1 | 7.449777 | 2.897197 | 0.048282 | up |
| LINC02060 | 3.615758 | 1.854298 | 0.031275 | up |
| GAPDHP35 | 4.802364 | 2.263745 | 0.030485 | up |
| SMIM15-AS1 | 2.872498 | 1.522306 | 0.024243 | up |
| AC005324.3 | 2.64456 | 1.403028 | 0.000999 | up |
| AC104825.2 | 1.655756 | 0.72749 | 0.003074 | up |
| HSPA8P13 | 6.36309 | 2.669727 | 0.005024 | up |
| AC067930.5 | 2.490884 | 1.316658 | 0.049308 | up |
| AL138752.2 | 19.54272 | 4.288559 | 0.02359 | up |
| LINC02421 | 7.954834 | 2.991832 | 0.036645 | up |
| MGAM | 3.140958 | 1.651205 | 5.18E-07 | up |
| MGAM2 | 3.786456 | 1.920848 | 0.001113 | up |
| AC083805.2 | 2.711022 | 1.438837 | 0.03757 | up |
| AL356019.2 | 1.625552 | 0.70093 | 0.011333 | up |
| TUBB3 | 3.629256 | 1.859674 | 0.027855 | up |
| NDUFA3P4 | 11.30013 | 3.498267 | 0.049224 | up |
| AC021739.2 | 6.052253 | 2.597472 | 0.02612 | up |
| AC020661.4 | 30.65121 | 4.937872 | 0.002172 | up |
| AC104794.2 | 1.779662 | 0.831604 | 0.041349 | up |
| AC034111.1 | 12.01786 | 3.587109 | 0.027497 | up |
| AL138781.1 | 1.771764 | 0.825186 | 0.01193 | up |
| AC022167.2 | 1.550351 | 0.632595 | 0.012839 | up |
| RBFADN | 1.682063 | 0.750232 | 0.038951 | up |
| AL360270.2 | 1.653938 | 0.725906 | 0.03997 | up |
| AC007220.1 | 4.364803 | 2.125917 | 0.004897 | up |
| SNORD3A | 1.751237 | 0.808374 | 0.006031 | up |
| MAGOH2P | 3.365652 | 1.750886 | 0.010953 | up |
| MIR3149 | 16.35577 | 4.031728 | 0.034797 | up |
| AC011195.2 | 2.793218 | 1.481928 | 0.044661 | up |
| AC007773.1 | 1.893888 | 0.921351 | 0.022499 | up |
| AC011481.2 | 16.31834 | 4.028422 | 0.034976 | up |
| LINC00868 | 16.31834 | 4.028422 | 0.034976 | up |
| RPL7P50 | 2.792466 | 1.48154 | 0.029849 | up |
| AL513477.1 | 2.157742 | 1.109523 | 8.00E-05 | up |
| MTDHP1 | 2.187261 | 1.129125 | 0.026347 | up |
| AC129492.5 | 21.69006 | 4.438962 | 0.01135 | up |
| AC091167.5 | 2.689328 | 1.427246 | 0.042419 | up |
| AC073389.2 | 20.27432 | 4.341581 | 0.026086 | up |
| AC022001.3 | 3.391087 | 1.761748 | 0.046753 | up |
| DGCR9 | 2.627722 | 1.393813 | 0.001556 | up |
| AP000692.2 | 1.747865 | 0.805594 | 0.015534 | up |
| AC004706.3 | 2.432938 | 1.2827 | 0.037561 | up |
| AC011815.2 | 2.210248 | 1.144208 | 0.000811 | up |
| LINC02340 | 1.928145 | 0.947213 | 0.000412 | up |
| FCGBP | 1.95493 | 0.967117 | 0.003591 | up |
| CU633906.1 | 2.935323 | 1.553519 | 0.016166 | up |
| CU639417.1 | 1.551512 | 0.633675 | 0.014356 | up |
| AC087392.5 | 3.327742 | 1.734543 | 0.039271 | up |
| AP003068.4 | 2.046116 | 1.032888 | 0.029121 | up |
| FP671120.1 | 3.634092 | 1.861595 | 0.003961 | up |
| AC015813.6 | 1.545959 | 0.628502 | 0.002686 | up |
| AP001324.3 | 12.99362 | 3.699732 | 0.026305 | up |
| AC131649.2 | 4.324131 | 2.11241 | 0.030107 | up |
| FAM238C | 1.77313 | 0.826299 | 0.042388 | up |
| AL034417.4 | 2.690588 | 1.427921 | 0.010455 | up |
| PDK4 | 0.518191 | -0.94844 | 0.013834 | down |
| HOXA11 | 0.639931 | -0.64401 | 0.006578 | down |
| ANLN | 0.622389 | -0.68411 | 0.000652 | down |
| TACC3 | 0.6237 | -0.68108 | 1.91E-07 | down |
| DEPDC1 | 0.586818 | -0.76902 | 4.70E-09 | down |
| HOXC8 | 0.549702 | -0.86328 | 1.68E-06 | down |
| ZIC2 | 0.623213 | -0.6822 | 0.000326 | down |
| PRDM6 | 0.629584 | -0.66753 | 0.003403 | down |
| ASPM | 0.619359 | -0.69115 | 0.046644 | down |
| BCL3 | 0.49249 | -1.02183 | 0.000731 | down |
| ARHGAP10 | 0.626276 | -0.67513 | 0.019704 | down |
| GTSE1 | 0.583902 | -0.7762 | 4.28E-07 | down |
| HOXA9 | 0.642994 | -0.63712 | 0.000242 | down |
| RUNX1T1 | 0.512542 | -0.96426 | 1.78E-05 | down |
| SLC4A4 | 0.617869 | -0.69463 | 0.049016 | down |
| NDC80 | 0.53252 | -0.90909 | 9.28E-09 | down |
| TRAF5 | 0.650694 | -0.61995 | 0.017056 | down |
| AURKA | 0.454784 | -1.13675 | 1.39E-14 | down |
| TPX2 | 0.572622 | -0.80435 | 1.91E-09 | down |
| KIF4A | 0.666285 | -0.58579 | 7.11E-06 | down |
| G2E3 | 0.588345 | -0.76527 | 0.00172 | down |
| NRP1 | 0.645099 | -0.63241 | 0.00837 | down |
| CARD10 | 0.650183 | -0.62108 | 0.015985 | down |
| DHRS2 | 0.250496 | -1.99714 | 6.32E-18 | down |
| MYLK2 | 0.497089 | -1.00843 | 0.000373 | down |
| FAM83D | 0.469104 | -1.09202 | 1.26E-18 | down |
| ELF4 | 0.641636 | -0.64017 | 0.000102 | down |
| FGF9 | 0.542396 | -0.88258 | 0.008284 | down |
| FOXF1 | 0.549335 | -0.86424 | 0.004459 | down |
| BMF | 0.459598 | -1.12156 | 1.31E-06 | down |
| HOXA1 | 0.561633 | -0.8323 | 8.88E-05 | down |
| HOXA3 | 0.627802 | -0.67162 | 0.001836 | down |
| GATA3 | 0.631116 | -0.66402 | 0.00272 | down |
| RNF43 | 0.565603 | -0.82214 | 4.12E-05 | down |
| WNT3 | 0.621632 | -0.68587 | 0.007517 | down |
| TRIM16L | 0.664265 | -0.59017 | 0.000432 | down |
| HOXB6 | 0.642036 | -0.63927 | 7.08E-05 | down |
| CDKN1B | 0.591434 | -0.75771 | 6.86E-06 | down |
| TBC1D30 | 0.616399 | -0.69806 | 0.002741 | down |
| CDCA3 | 0.532529 | -0.90907 | 6.75E-06 | down |
| BMP5 | 0.359185 | -1.4772 | 0.011092 | down |
| RBM24 | 0.512161 | -0.96533 | 0.026891 | down |
| SOBP | 0.625821 | -0.67618 | 0.005952 | down |
| TTK | 0.598591 | -0.74036 | 1.02E-06 | down |
| KIF20A | 0.43535 | -1.19975 | 3.11E-20 | down |
| WNT5A | 0.591743 | -0.75696 | 0.008013 | down |
| FOXP1 | 0.663474 | -0.59189 | 0.025148 | down |
| CENPA | 0.419772 | -1.25232 | 3.12E-07 | down |
| CDC20 | 0.501489 | -0.99571 | 1.47E-08 | down |
| CENPF | 0.612038 | -0.70831 | 0.015465 | down |
| PHF3 | 0.642705 | -0.63777 | 0.018343 | down |
| IRF2BPL | 0.659039 | -0.60156 | 0.001263 | down |
| HOXB5 | 0.574342 | -0.80002 | 0.00014 | down |
| DUSP4 | 0.554874 | -0.84977 | 0.014838 | down |
| TMEM131L | 0.615232 | -0.7008 | 0.000776 | down |
| KCNJ8 | 0.621458 | -0.68627 | 0.00137 | down |
| SLITRK3 | 0.534467 | -0.90383 | 0.002268 | down |
| HOXC13 | 0.651601 | -0.61794 | 0.017754 | down |
| HOXC11 | 0.636114 | -0.65264 | 0.011006 | down |
| HJURP | 0.481373 | -1.05477 | 3.79E-16 | down |
| CKS2 | 0.560072 | -0.83632 | 0.000154 | down |
| CTCFL | 0.125576 | -2.99337 | 0.039798 | down |
| RUNX2 | 0.499528 | -1.00136 | 0.035015 | down |
| SOX21 | 0.498105 | -1.00548 | 0.018275 | down |
| NKX2-4 | 0.52823 | -0.92076 | 8.31E-05 | down |
| DLGAP5 | 0.548383 | -0.86674 | 1.13E-07 | down |
| BCL11B | 0.560787 | -0.83447 | 0.004841 | down |
| LRFN1 | 0.658897 | -0.60187 | 0.008871 | down |
| HOXD3 | 0.173913 | -2.52356 | 0.014603 | down |
| GAD1 | 0.54689 | -0.87068 | 5.13E-05 | down |
| TOP2A | 0.549295 | -0.86435 | 0.000579 | down |
| CCNB1 | 0.535126 | -0.90205 | 1.04E-10 | down |
| MEIS2 | 0.640176 | -0.64346 | 1.25E-05 | down |
| PSRC1 | 0.401981 | -1.3148 | 7.57E-09 | down |
| CDCA8 | 0.528471 | -0.9201 | 4.67E-13 | down |
| PRRG4 | 0.522908 | -0.93537 | 0.001588 | down |
| ESPL1 | 0.662215 | -0.59463 | 0.001035 | down |
| THSD1 | 0.619309 | -0.69127 | 0.040708 | down |
| GATA4 | 0.664272 | -0.59015 | 0.001276 | down |
| IL36G | 0.099011 | -3.33626 | 0.013619 | down |
| FOXF2 | 0.560579 | -0.83501 | 0.001108 | down |
| KIF23 | 0.490594 | -1.0274 | 1.96E-15 | down |
| SEMA6D | 0.610493 | -0.71195 | 0.009428 | down |
| CYP1B1 | 0.520888 | -0.94095 | 2.45E-07 | down |
| FGF5 | 0.619228 | -0.69146 | 0.02528 | down |
| CENPE | 0.537274 | -0.89627 | 0.003749 | down |
| PRICKLE1 | 0.601232 | -0.73401 | 0.001541 | down |
| GAS2L3 | 0.591226 | -0.75822 | 0.002661 | down |
| PDX1 | 0.648907 | -0.62392 | 0.008559 | down |
| PIF1 | 0.296175 | -1.75548 | 1.10E-13 | down |
| CYP1A1 | 0.377395 | -1.40585 | 3.65E-09 | down |
| TICRR | 0.663423 | -0.592 | 0.014762 | down |
| HUNK | 0.601949 | -0.73229 | 0.002366 | down |
| TINAGL1 | 0.110664 | -3.17574 | 0.022825 | down |
| KIF2C | 0.610163 | -0.71273 | 9.06E-07 | down |
| MEIS1 | 0.633781 | -0.65794 | 3.18E-05 | down |
| DLX1 | 0.61796 | -0.69441 | 7.59E-06 | down |
| EPHA5 | 0.591191 | -0.7583 | 0.031636 | down |
| SSBP2 | 0.637941 | -0.64851 | 0.001293 | down |
| VLDLR | 0.62243 | -0.68402 | 0.035733 | down |
| MKI67 | 0.551236 | -0.85926 | 0.003114 | down |
| HTR3B | 0.436686 | -1.19533 | 0.03159 | down |
| INCENP | 0.572822 | -0.80384 | 0.001312 | down |
| ARID5B | 0.532638 | -0.90877 | 0.00051 | down |
| LATS2 | 0.64433 | -0.63413 | 0.014626 | down |
| PRDM8 | 0.441145 | -1.18068 | 0.000523 | down |
| BUB1B | 0.557125 | -0.84393 | 0.003283 | down |
| CLEC18C | 0.3055 | -1.71076 | 0.049069 | down |
| KIT | 0.47577 | -1.07166 | 0.000995 | down |
| CCNB2 | 0.593543 | -0.75258 | 3.77E-06 | down |
| DUSP2 | 0.508945 | -0.97442 | 0.04309 | down |
| AAED1 | 0.599107 | -0.73912 | 0.005869 | down |
| DNAH3 | 0.245011 | -2.02908 | 1.96E-08 | down |
| ZFAND2B | 0.635403 | -0.65426 | 0.005029 | down |
| TUBBP5 | 0.611597 | -0.70935 | 0.002996 | down |
| IRX6 | 0.503356 | -0.99035 | 0.003336 | down |
| GAB3 | 0.322232 | -1.63383 | 0.032004 | down |
| RACGAP1 | 0.530966 | -0.91331 | 2.95E-09 | down |
| CCNF | 0.528949 | -0.9188 | 2.67E-11 | down |
| NFIA | 0.617591 | -0.69528 | 0.001977 | down |
| LHX8 | 0.426306 | -1.23004 | 5.49E-05 | down |
| GBP2 | 0.386645 | -1.37092 | 0.015507 | down |
| SGO2 | 0.611986 | -0.70843 | 0.002084 | down |
| TIPARP | 0.547574 | -0.86887 | 1.08E-05 | down |
| SPRY1 | 0.616612 | -0.69756 | 0.009999 | down |
| GUCY1A3 | 0.643133 | -0.63681 | 0.027064 | down |
| EBF1 | 0.601962 | -0.73226 | 0.024837 | down |
| TLX3 | 0.570669 | -0.80927 | 0.023448 | down |
| BMPER | 0.556324 | -0.846 | 0.014609 | down |
| SP8 | 0.520545 | -0.94191 | 2.45E-05 | down |
| AL353743.1 | 0.560533 | -0.83513 | 0.005431 | down |
| CDX2 | 0.566919 | -0.81879 | 1.13E-07 | down |
| DACT1 | 0.523382 | -0.93406 | 0.001466 | down |
| PLK1 | 0.409913 | -1.28661 | 4.36E-24 | down |
| RCOR2 | 0.661729 | -0.59569 | 0.029491 | down |
| TTC39C | 0.650528 | -0.62032 | 0.00109 | down |
| ATOH8 | 0.451623 | -1.14681 | 0.007583 | down |
| ZNF608 | 0.507907 | -0.97736 | 0.001451 | down |
| PXDC1 | 0.474571 | -1.0753 | 0.003476 | down |
| CKAP2L | 0.585749 | -0.77165 | 0.000445 | down |
| BUB1 | 0.632581 | -0.66068 | 8.27E-05 | down |
| CDK1 | 0.660133 | -0.59917 | 5.73E-05 | down |
| EMX2 | 0.658717 | -0.60227 | 0.006747 | down |
| AC093323.1 | 0.475565 | -1.07228 | 6.48E-12 | down |
| ENC1 | 0.63925 | -0.64555 | 0.005647 | down |
| FOXB1 | 0.507614 | -0.9782 | 0.030312 | down |
| INSM1 | 0.32472 | -1.62273 | 0.000725 | down |
| PHLDA3 | 0.575152 | -0.79799 | 0.017301 | down |
| GALNTL6 | 0.561848 | -0.83175 | 0.045918 | down |
| IL20RB | 0.548408 | -0.86668 | 0.021106 | down |
| UBE2C | 0.403129 | -1.31069 | 7.58E-10 | down |
| NR2F1 | 0.643658 | -0.63563 | 1.51E-05 | down |
| RNF152 | 0.561465 | -0.83273 | 0.011232 | down |
| FOXC2 | 0.476143 | -1.07053 | 0.010777 | down |
| IRX5 | 0.640092 | -0.64365 | 0.002311 | down |
| TGIF1 | 0.488939 | -1.03227 | 1.20E-08 | down |
| IRX3 | 0.609323 | -0.71472 | 0.032734 | down |
| RPRM | 0.493019 | -1.02028 | 0.002151 | down |
| CCDC184 | 0.603249 | -0.72917 | 0.02907 | down |
| NEUROG2 | 0.32966 | -1.60095 | 0.003441 | down |
| AURKB | 0.510713 | -0.96942 | 1.42E-07 | down |
| C3orf80 | 0.508862 | -0.97465 | 0.000792 | down |
| ALX1 | 0.612481 | -0.70726 | 0.003069 | down |
| MB21D2 | 0.608834 | -0.71588 | 3.65E-05 | down |
| MAB21L1 | 0.597432 | -0.74315 | 0.000294 | down |
| SHISA2 | 0.598728 | -0.74003 | 0.000859 | down |
| HOXC9 | 0.638757 | -0.64666 | 0.001142 | down |
| SOX2 | 0.608303 | -0.71714 | 0.000277 | down |
| KPNA2 | 0.557991 | -0.84169 | 1.25E-11 | down |
| BCOR | 0.663216 | -0.59245 | 0.009443 | down |
| RIPK4 | 0.560063 | -0.83634 | 1.67E-07 | down |
| CCR4 | 0.172979 | -2.53133 | 0.02562 | down |
| CSF1 | 0.539128 | -0.8913 | 0.009877 | down |
| TMEM173 | 0.662373 | -0.59428 | 0.038749 | down |
| CDCA2 | 0.570481 | -0.80975 | 0.000296 | down |
| PRSS57 | 0.088608 | -3.49641 | 0.040984 | down |
| UBALD2 | 0.642063 | -0.63921 | 0.031143 | down |
| PTCH1 | 0.587917 | -0.76631 | 0.009444 | down |
| KIF18B | 0.588257 | -0.76548 | 3.11E-09 | down |
| MYT1L | 0.362936 | -1.46221 | 0.024752 | down |
| FOXD2 | 0.664515 | -0.58963 | 0.006375 | down |
| FOXD3 | 0.593796 | -0.75196 | 0.016128 | down |
| ARHGAP11B | 0.643749 | -0.63543 | 0.008238 | down |
| FAM72B | 0.461319 | -1.11616 | 2.42E-14 | down |
| FAM72A | 0.43503 | -1.20081 | 5.40E-10 | down |
| MIRLET7BHG | 0.440529 | -1.18269 | 0.037514 | down |
| NOL4L | 0.630921 | -0.66447 | 0.017597 | down |
| C6orf141 | 0.59155 | -0.75743 | 0.040019 | down |
| MAP3K5 | 0.616267 | -0.69837 | 0.030043 | down |
| PDGFA | 0.526752 | -0.9248 | 0.011479 | down |
| KLHL14 | 0.477923 | -1.06515 | 0.025196 | down |
| HOXC6 | 0.656757 | -0.60657 | 0.005414 | down |
| ARHGAP11A | 0.574688 | -0.79915 | 0.000212 | down |
| PRC1 | 0.622423 | -0.68403 | 3.60E-07 | down |
| RN7SKP78 | 0.166349 | -2.58772 | 0.03606 | down |
| SNORD67 | 0.329465 | -1.6018 | 0.041805 | down |
| FAM72D | 0.44072 | -1.18206 | 6.27E-11 | down |
| RNU4-78P | 0.068635 | -3.86492 | 0.049515 | down |
| AC106786.1 | 0.627183 | -0.67304 | 0.015095 | down |
| LINC01529 | 0.197875 | -2.33734 | 0.018548 | down |
| AL158834.2 | 0.045481 | -4.45859 | 0.013746 | down |
| LINC01068 | 0.068786 | -3.86173 | 0.049756 | down |
| MIR181A1HG | 0.068786 | -3.86173 | 0.049756 | down |
| AC092813.2 | 0.417151 | -1.26136 | 0.031931 | down |
| STMND1 | 0.247665 | -2.01354 | 0.002961 | down |
| AP000253.1 | 0.44025 | -1.18361 | 0.014322 | down |
| ESRRAP2 | 0.330783 | -1.59604 | 0.023559 | down |
| AC011247.2 | 0.640785 | -0.64209 | 0.04199 | down |
| LINC01647 | 0.370107 | -1.43399 | 0.027026 | down |
| NR2F1-AS1 | 0.604683 | -0.72575 | 0.003739 | down |
| KIFC1 | 0.459544 | -1.12173 | 6.12E-12 | down |
| AL109615.3 | 0.386201 | -1.37258 | 0.000242 | down |
| POM121L7P | 0.061347 | -4.02687 | 0.040932 | down |
| AC123768.1 | 0.363876 | -1.45848 | 0.027105 | down |
| ALG1L15P | 0.068635 | -3.86492 | 0.049515 | down |
| AC093663.2 | 0.055117 | -4.18135 | 0.031292 | down |
| HULC | 0.054879 | -4.18761 | 0.024847 | down |
| WWC2-AS2 | 0.290337 | -1.7842 | 0.036124 | down |
| RNU6-781P | 0.048703 | -4.35985 | 0.004554 | down |
| LINC01605 | 0.599336 | -0.73856 | 0.022994 | down |
| AP001189.5 | 0.241192 | -2.05175 | 0.000497 | down |
| HSPD1P4 | 0.476064 | -1.07077 | 0.029331 | down |
| SPESP1 | 0.114168 | -3.13077 | 0.009633 | down |
| AC009269.5 | 0.350096 | -1.51418 | 0.022154 | down |
| AC012435.1 | 0.336862 | -1.56977 | 0.000154 | down |
| AC104083.1 | 0.603428 | -0.72875 | 0.033349 | down |
| DOCK9-AS2 | 0.305806 | -1.70931 | 0.023871 | down |
| AC133919.2 | 0.624504 | -0.67922 | 0.044654 | down |
| AC026992.2 | 0.097322 | -3.3611 | 0.049281 | down |
| FAM72C | 0.417349 | -1.26067 | 1.54E-06 | down |
| AC032019.1 | 0.067372 | -3.89171 | 0.020047 | down |
| RAB11B-AS1 | 0.420152 | -1.25102 | 0.016952 | down |
| AC091057.3 | 0.282076 | -1.82585 | 0.001523 | down |
| AL391358.1 | 0.209826 | -2.25273 | 0.00011 | down |
| AC016575.1 | 0.097232 | -3.36242 | 0.046132 | down |
| AL022238.3 | 0.149063 | -2.74601 | 0.034029 | down |
| AC009269.6 | 0.324469 | -1.62385 | 0.029876 | down |
| AC005332.7 | 0.619092 | -0.69177 | 0.042269 | down |
| MIR6768 | 0.097534 | -3.35795 | 0.049752 | down |
| AL031710.2 | 0.640822 | -0.64201 | 0.031803 | down |
| PRAG1 | 0.63572 | -0.65354 | 0.027931 | down |
| LINC01667 | 0.357156 | -1.48537 | 0.036916 | down |
| AC026401.3 | 0.579186 | -0.7879 | 0.007125 | down |
| FOXCUT | 0.420221 | -1.25078 | 0.000765 | down |
| AL732314.6 | 0.034147 | -4.87211 | 0.014653 | down |
